# The genome sequence of Water Lobelia, *Lobelia dortmanna* L., 1753 (Asterales: Campanulaceae)

**DOI:** 10.12688/wellcomeopenres.27537.1

**Published:** 2026-10-08

**Authors:** Maarten J. M. Christenhusz, Ilia J. Leitch, Michael F. Fay, Royal Botanic Gardens Kew Genome Acquisition Lab, Plant Genome Sizing Collective, Darwin Tree of Life Barcoding collective, Wellcome Sanger Institute Tree of Life Management, Samples and Laboratory team, Wellcome Sanger Institute Scientific Operations: Sequencing Operations, Wellcome Sanger Institute Tree of Life Core Informatics team, Tree of Life Core Informatics collective, Darwin Tree of Life Consortium

**Affiliations:** 1 Royal Botanic Gardens Kew, Richmond, England, UK; 2 Botanical Society of Britain and Ireland, St Albans, Hertfordshire, England, UK

**Keywords:** Lobelia dortmanna, Water Lobelia, genome sequence, chromosomal, Asterales

## Abstract

We present a genome assembly of *Lobelia dortmanna* (Water Lobelia; Streptophyta; Magnoliopsida; Asterales; Campanulaceae). The assembly consists of two haplotypes with total lengths of 780.10 megabases and 756.83 megabases. Most of haplotype 1 (99.63%) is scaffolded into 7 chromosomal pseudomolecules. Haplotype 2 was assembled to scaffold level. The mitochondrial sequence has a length of 496.36 kilobases and the plastid genome assembly has a length of 163.59 kilobases. This assembly was generated as part of the Darwin Tree of Life project, which produces reference genomes for eukaryotic species found in Britain and Ireland.

## Species taxonomy

Eukaryota; Viridiplantae; Streptophyta; Streptophytina; Embryophyta; Tracheophyta; Euphyllophyta; Spermatophyta; Magnoliopsida; Mesangiospermae; eudicotyledons; Gunneridae; Pentapetalae; asterids; campanulids; Asterales; Campanulaceae; *Lobelia*; *Lobelia dortmanna* L., 1753 (NCBI:txid368684).

## Background

As part of the Darwin Tree of Life Project, a collaborative effort to sequence all named eukaryotic species in the Atlantic Archipelago of Britain and Ireland, we sequenced the genome of water lobelia, *Lobelia dortmanna* L. Here we present a chromosomally complete genome sequence based on a wild specimen from Llyn y Dywarchen near Rhys-Ddu, Gwynedd, Wales, UK.

*Lobelia* L. is a large cosmopolitan genus with over 400 species, as currently circumscribed (e.g. Lammers, 2011). In this circumscription it has been shown to be grossly polyphyletic, and taxonomic revision will be required to achieve monophyly (e.g. Antonelli, 2008; Kagame *et al.*, 2021). Nevertheless, as *Lobelia dortmanna* is the type species of the genus, its name will remain unchanged even if the decision is made to split *Lobelia.* The genus was named in honour of the Flemish botanist and physician Matthias de l’Obel (1538–1616), whose name was Latinised as Matthaeus Lobelius. The epithet is named for Dutch apothecary Jan Dortmann, a contemporary of Lobelius.

*Lobelia dortmanna* ( Figure 1) is a small perennial herbaceous aquatic plant, growing usually submerged but sometimes temporarily exposed on lake edges. The strap-like leaves form a rosette, from which emerge lax-flowering stems, up to 70 cm tall (occasionally to 120 cm), bearing three to ten flowers above the surface of the water. The flowers are pale lilac ( Stace, 2010).

*Lobelia dortmanna* occurs widely in northern and western Europe (from France to northern Norway) and in North America ( POWO, 2026), growing in gravelly, stony or silty acidic substrates in nutrient-poor water. It is slow growing and restricted to shallow water due to its inability to grow and compete under low light conditions in deeper water ( Farmer & Spence, 1987). In Britain and Ireland, it is widespread in suitable habitats, predominantly in north-western Britain and western Ireland. It occurs at elevations up to 745 m ( BSBI, 2026).

The sporophytic chromosome count has been reported to be 2 *n* = 14 (e.g. Henniges *et al.*, 2022). Most of the genome assembly is scaffolded into seven chromosomal pseudomolecules, reflecting the gametophytic chromosome count of this species (i.e. *n* = 7).

This genome, in combination with genomes of other members of Campanulaceae subfamily Lobelioideae (e.g. Zhang *et al.*, 2025), will provide a valuable resource for comparative genomics and a foundation for a stable nomenclatural treatment of the group.

## Methods

### Sample acquisition, flow cytometry and DNA barcoding

A specimen of *L. dortmanna* (specimen ID KDTOL10333, ToLID daLobDort1) was used for genome sequencing. It was collected from Llyn y Dywarchen, Gwynedd, Wales, UK (latitude 53.0576, longitude −4.1502) on 2021-08-12. The specimen was collected and identified by Maarten Christenhusz. The daLobDort1 specimen was also used for Hi-C and RNA sequencing. The herbarium voucher associated with the sequenced plant is 9251 and is deposited in the herbarium of RBG Kew (K) ( K001400917).

The genome size was estimated by flow cytometry following the ‘one-step’ method outlined in Pellicer *et al*. (2021) and using propidium iodide as the fluorochrome. The General Purpose Buffer (GPB) supplemented with 3% PVP was used for isolation of nuclei ( Loureiro *et al.*, 2007), and the internal calibration standard was *Petroselinum crispum* ‘Champion Moss Curled’ with an assumed 1C-value of 2,200 Mb ( Obermayer *et al.*, 2002).

The initial identification was verified by an additional DNA barcoding process according to the framework developed by Twyford *et al*. (2024). Part of the plant specimen was preserved in silica gel desiccant ( Chase & Hills, 1991). DNA extracted from the dried plant was amplified by PCR for standard barcode markers, with the amplicons sequenced and compared to public sequence databases including GenBank and the Barcode of Life Database (BOLD) ( Ratnasingham & Hebert, 2007). Following whole genome sequence generation, the relevant DNA barcode region was also used alongside the initial barcoding data for sample tracking at the WSI ( Twyford *et al.*, 2024). The standard operating procedures for Darwin Tree of Life barcoding are available on protocols.io.

### Nucleic acid extraction

High molecular weight (HMW) DNA extraction protocols developed by the Tree of Life Core Laboratory at the Wellcome Sanger Institute (WSI) are available on protocols.io ( Howard *et al.*, 2025). Tissue from the daLobDort1 sample was weighed and triaged to select the appropriate extraction workflow. HMW DNA was prepared for one PacBio HiFi library: DTOL13810556. Leaf tissue was disrupted using the Covaris cryoPREP method. DNA was extracted using the Automatic Plant Organic Extraction protocol, and DNA was fragmented following the Sanger Tree of Life HMW DNA Fragmentation: Covaris g-TUBE for ULI PacBio protocol. The sample was cleaned-up by Automated SPRI. The concentration of the sheared and purified DNA was assessed using a NanoDrop spectrophotometer and Qubit Fluorometer using the Qubit dsDNA High Sensitivity Assay kit. Fragment size distribution was evaluated by running the sample on the FemtoPulse system. For this sample, final post-shearing DNA measurements included a Qubit concentration of 2.38 ng/μL, and an average fragment size of 9.4 kb.

RNA was extracted from leaf tissue of daLobDort1 in the Tree of Life Laboratory at the WSI using the RNA Extraction: Automated MagMax™ *mir*Vana protocol. The RNA concentration was assessed using a NanoDrop spectrophotometer and a Qubit Fluorometer using the Qubit RNA Broad-Range Assay kit. Analysis of the integrity of the RNA was done using the Agilent RNA 6000 Pico Kit and Eukaryotic Total RNA assay.

### PacBio HiFi library preparation and sequencing

Library preparation and sequencing were performed at the WSI Scientific Operations core. Library DTOL13810556 was prepared using the recorded ultra-low-input PacBio HiFi workflow. Prior to library preparation, the DNA was fragmented to ~10 kb. Ultra-low-input (ULI) libraries were prepared using the PacBio SMRTbell ^®^ Express Template Prep Kit 2.0 and gDNA Sample Amplification Kit. Samples were normalised to 20 ng DNA. Single-strand overhang removal, DNA damage repair, and end-repair/A-tailing were performed according to the manufacturer’s instructions, followed by adapter ligation. A 0.85× pre-PCR clean-up was carried out with Promega ProNex beads.

The DNA was evenly divided into two aliquots for dual PCR (reactions A and B), both following the manufacturer’s protocol. A 0.85× post-PCR clean-up was performed with ProNex beads. DNA concentration was measured using a Qubit Fluorometer v4.0 (Thermo Fisher Scientific) with the Qubit HS Assay Kit, and fragment size was assessed on an Agilent Femto Pulse Automated Pulsed Field CE Instrument (Agilent Technologies) using the gDNA 55 kb BAC analysis kit. PCR reactions A and B were then pooled, ensuring a total mass of ≥500 ng in 47.4 μl.

The pooled sample underwent another round of DNA damage repair, end-repair/A-tailing, and hairpin adapter ligation. A 1× clean-up was performed with ProNex beads, followed by DNA quantification using the Qubit and fragment size analysis using the Agilent Femto Pulse. Size selection was performed on the Sage Sciences PippinHT system, with target fragment size determined by Femto Pulse analysis (typically 4–9 kb). Size-selected libraries were cleaned with 1.0× ProNex beads and normalised to 2 nM before sequencing.

Library DTOL13810556 was sequenced on a Revio instrument (Pacific Biosciences, California, USA). Prepared libraries with a molarity greater than 2 nM were normalised to 2 nM, and 15 μL was used for making complexes. For libraries with a molarity below 2 nM, the available 10 μL library volume was used without normalisation. Primers were annealed and polymerases were bound to generate circularised complexes, following the manufacturer’s instructions. Complexes were purified using 1.2× SMRTbell beads, diluted to the Revio loading concentration of 200–300 pM, and spiked with a Revio sequencing internal control. Sequencing used Revio 25 M SMRT cells. SMRT Link software (Pacific Biosciences), a web-based workflow manager, was used to configure and monitor the run and to carry out primary and secondary data analysis.

### Hi-C

***Sample preparation and crosslinking***

Hi-C data were generated from 50 mg of the daLobDort1 leaf sample using the Arima-HiC v2 kit (Arima Genomics). Tissue was finely ground using the Covaris cryoPREP Dry Pulverizer (Covaris), and then subjected to nuclei isolation. Nuclei were isolated using a modified protocol based on the Qiagen QProteome Cell Compartment Kit (Qiagen), in which only the Lysis and CE2 buffers were used, with QIAshredder spin columns. After isolation, nuclei were fixed using formaldehyde to a final concentration of 2% to crosslink the DNA. The crosslinked DNA was then digested and biotinylated according to the manufacturer’s instructions. A clean-up step was performed with SPRIselect beads before library preparation. DNA concentration was quantified using the Qubit Fluorometer v4.0 (Thermo Fisher Scientific) and the Qubit HS Assay Kit, following the manufacturer’s instructions.

***Hi-C library preparation and sequencing***

Biotinylated DNA constructs were fragmented using a Covaris E220 sonicator and size selected to 400–600 bp using SPRISelect beads. DNA was enriched with Arima-HiC v2 kit Enrichment beads. End repair, A-tailing, and adapter ligation were carried out with the NEBNext Ultra II DNA Library Prep Kit (New England Biolabs), following a modified protocol where library preparation occurs while DNA remains bound to the Enrichment beads. Library amplification was performed using KAPA HiFi HotStart mix and a custom Unique Dual Index (UDI) barcode set (Integrated DNA Technologies). Depending on sample concentration and biotinylation percentage determined at the crosslinking stage, libraries were amplified with 10–16 PCR cycles. Post-PCR clean-up was performed with SPRISelect beads. Libraries were quantified using the AccuClear Ultra High Sensitivity dsDNA Standards Assay Kit (Biotium) and a FLUOstar Omega plate reader (BMG Labtech).

Prior to sequencing, libraries were normalised to 10 ng/μL. Normalised libraries were quantified again to create equimolar and/or weighted 2.8 nM pools. Pool concentrations were checked using the Agilent 4200 TapeStation (Agilent) with High Sensitivity D500 reagents before sequencing. Sequencing was performed using paired-end 150 bp reads on the Illumina NovaSeq 6000.

### RNA library preparation and sequencing

Libraries were prepared using the NEBNext ^®^ Ultra II Directional RNA Library Prep Kit for Illumina (New England Biolabs), following the manufacturer’s instructions. Poly(A) mRNA was isolated from 100 ng of total RNA using oligo (dT) beads, converted to cDNA, and uniquely indexed; 14 PCR cycles were performed. Libraries were prepared with an intended average fragment size of 100–300 bp. Libraries were quantified, normalised, and pooled equimolarly to a final concentration of 2.8 nM before dilution to 150 pM for loading. Sequencing was carried out on the Illumina NovaSeq 6000, generating paired-end reads.

### Genome assembly

Prior to assembly of the PacBio HiFi reads, a database of *k*-mer counts ( *k* = 31) was generated from the filtered reads using FastK. GenomeScope2 ( Ranallo-Benavidez *et al.*, 2020) was used to analyse the *k*-mer frequency distributions, providing estimates of genome size, heterozygosity, and repeat content.

The HiFi reads were assembled using Hifiasm in Hi-C phasing mode ( Cheng *et al.*, 2021, 2022), producing two haplotypes. Hi-C reads ( Rao *et al.*, 2014) were mapped to the primary contigs using bwa-mem2 ( Vasimuddin *et al.*, 2019). Contigs were further scaffolded with Hi-C data in YaHS ( Zhou *et al.*, 2023), using the --break option for handling potential misassemblies. The scaffolded assemblies were evaluated using Gfastats ( Formenti *et al.*, 2022), BUSCO ( Manni *et al.*, 2021) and MerquryFK ( Rhie *et al.*, 2020). The organelle genomes were assembled using OATK ( Zhou *et al.*, 2025). The comparator for the mitochondrial assembly was the mitochondrial genome of *Codonopsis lanceolata* (NC_037949.1).

### Assembly curation

The assembly was decontaminated using the Assembly Screen for Cobionts and Contaminants ( ASCC) pipeline. TreeVal was used to generate the flat files and maps for use in curation. Manual curation was conducted primarily in PretextView and HiGlass ( Kerpedjiev *et al.*, 2018). Scaffolds were visually inspected and corrected as described by Howe *et al*. (2021). Manual corrections included eight breaks and 11 joins. This reduced the scaffold count by 98.5% and reduced the total assembly length by 7.4%. The curation process is described at sanger-tol/curation-resources . PretextSnapshot was used to generate a Hi-C contact map of the final assembly.

### Assembly quality assessment

The MerquryFK tool ( Rhie *et al.*, 2020) was run in a Singularity container ( Kurtzer *et al.*, 2017) to evaluate *k*-mer completeness and assembly quality for both haplotypes using the *k*-mer database ( *k* = 31) computed prior to genome assembly. The analysis outputs included assembly QV scores and completeness statistics.

The genome was analysed using the BlobToolKit pipeline, a Nextflow implementation of the earlier Snakemake version ( Challis *et al.*, 2020). PacBio reads were aligned to the assembly using minimap2 ( Li, 2018) and processed with SAMtools ( Danecek *et al.*, 2021) to generate coverage tracks. The pipeline runs BUSCO ( Manni *et al.*, 2021) using lineages identified from the NCBI Taxonomy ( Schoch *et al.*, 2020). For the three basal BUSCO lineages, eukaryota, bacteria and archaea, BUSCO genes are aligned to the UniProt Reference Proteomes database ( Bateman *et al.*, 2023) using DIAMOND BLASTp ( Buchfink *et al.*, 2021). The genome assembly is divided into chunks based on the density of BUSCO genes from the closest taxonomic lineage, and each chunk is aligned to the UniProt Reference Proteomes database using DIAMOND BLASTx. Sequences without DIAMOND BLASTx hits are extracted using seqtk and aligned to the NT database using BLASTn ( Altschul *et al.*, 1990). The outputs are consolidated into a BlobDir for visualisation in BlobToolKit. The BlobToolKit pipeline was developed using nf-core tooling ( Ewels *et al.*, 2020) and MultiQC ( Ewels *et al.*, 2016), with containerisation through Docker ( Merkel, 2014) and Singularity ( Kurtzer *et al.*, 2017).

## Genome sequence report

### Sequence data

The genome of a specimen of *L. dortmanna* was sequenced using Pacific Biosciences single-molecule HiFi long reads, generating 79.67 Gb (gigabases) from 9.65 million reads, which were used to assemble the genome. GenomeScope2.0 analysis using a *p* = 2 model gave a *k*-mer-based genome size estimate of 915.17 Mb, with a heterozygosity of 0.76% and repeat content of 57.33% ( Figure 2). Using flow cytometry, the genome size (1C-value) of the sample was estimated to be 1.11 pg, equivalent to 1,080.00 Mb. These estimates guided expectations for the assembly. Based on the estimated genome size, the sequencing data provided approximately 75× coverage. Hi-C sequencing produced 131.98 Gb from 437.02 million read pairs, which were used to scaffold the assembly. RNA sequencing data were also generated and are available in public sequence repositories. Table 1 summarises the specimen and sequencing details.

**Figure 1.**
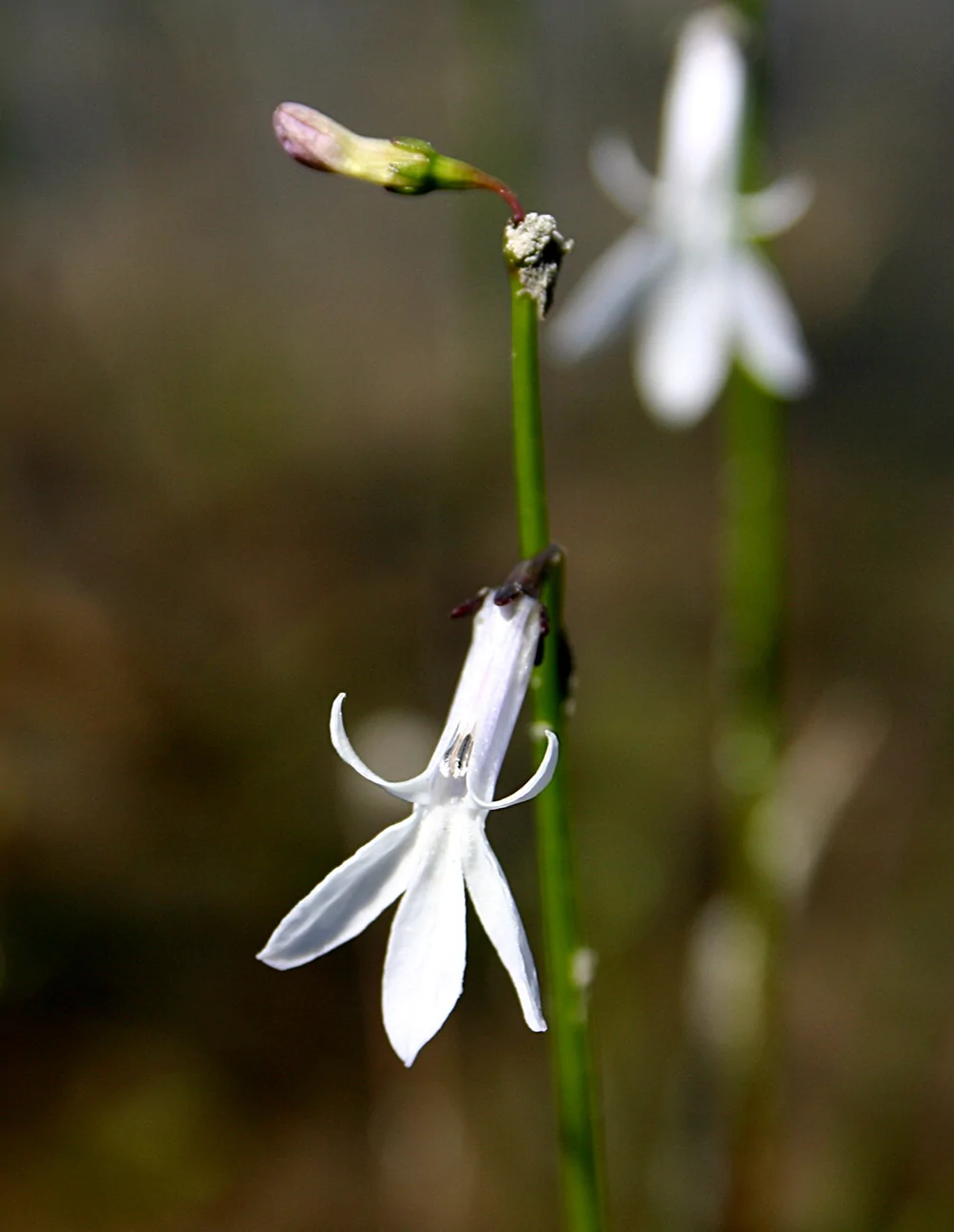
Photograph of *L. dortmanna* by Przykuta.

**Table 1.** Specimen and sequencing data for *L. dortmanna* (BioProject PRJEB79878).

Platform	PacBio HiFi	Hi-C	RNA-seq
**ToLID**	daLobDort1	daLobDort1	daLobDort1
**Specimen ID**	KDTOL10333	KDTOL10333	KDTOL10333
**BioSample (source individual)**	SAMEA10369529	SAMEA10369529	SAMEA10369529
**BioSample (tissue)**	SAMEA10369600	SAMEA10369600	SAMEA10369600
**Tissue**	leaf	leaf	leaf
**Instrument**	Revio	Illumina NovaSeq 6000	Illumina NovaSeq 6000
**Library ID & run accession(s)**	DTOL13810556: ERR13660052	ERR13670009	ERR13670010
**Read count total**	9.65 million reads	437.02 million read pairs	22.83 million read pairs
**Base count total**	79.67 Gb	131.98 Gb	6.89 Gb

**Figure 2.**
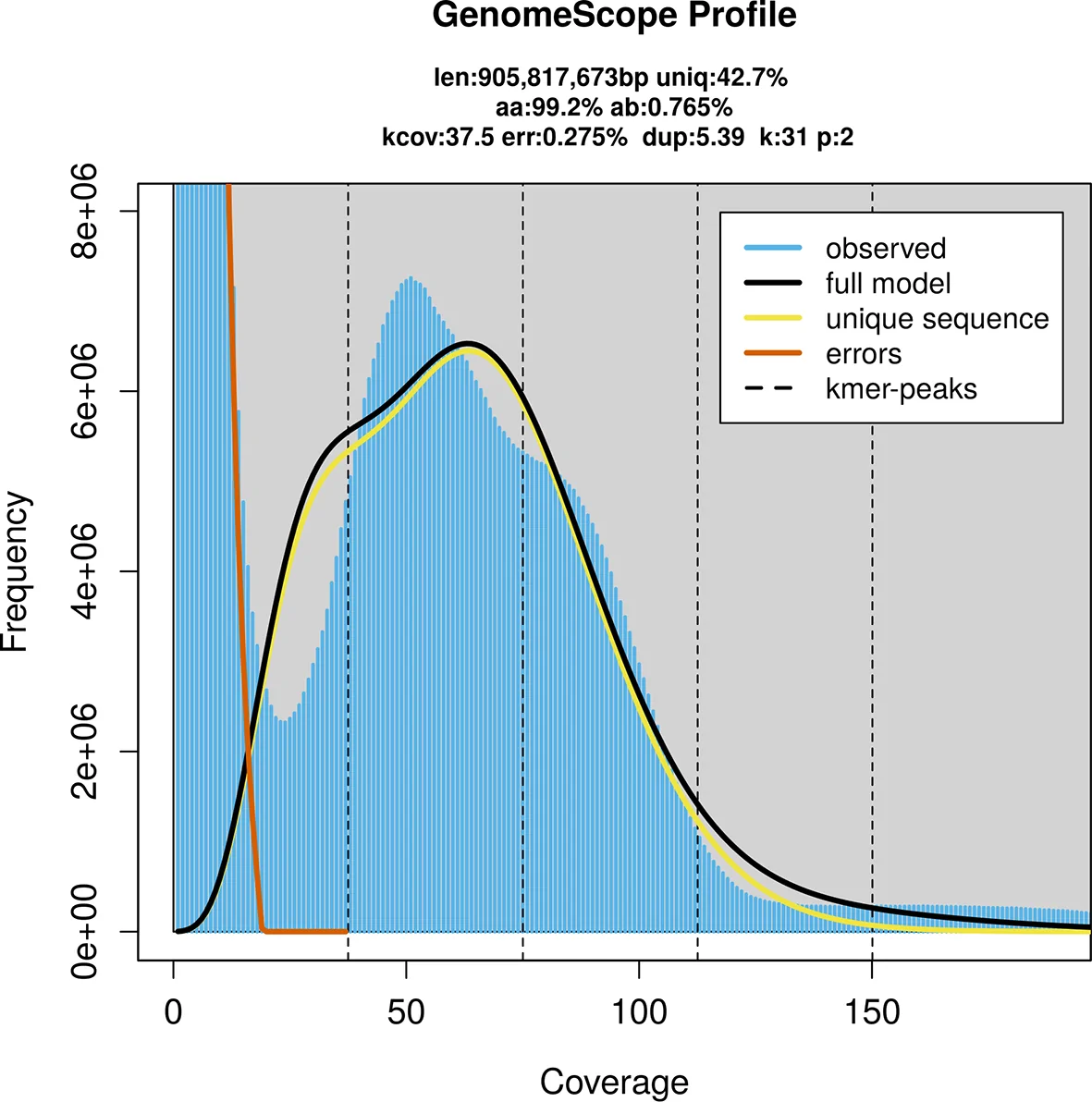
Frequency distribution of *k*-mers generated using GenomeScope2. The plot shows observed and modelled *k*-mer spectra, providing estimates of genome size, heterozygosity, and repeat content based on unassembled sequencing reads.

**Figure 3.**
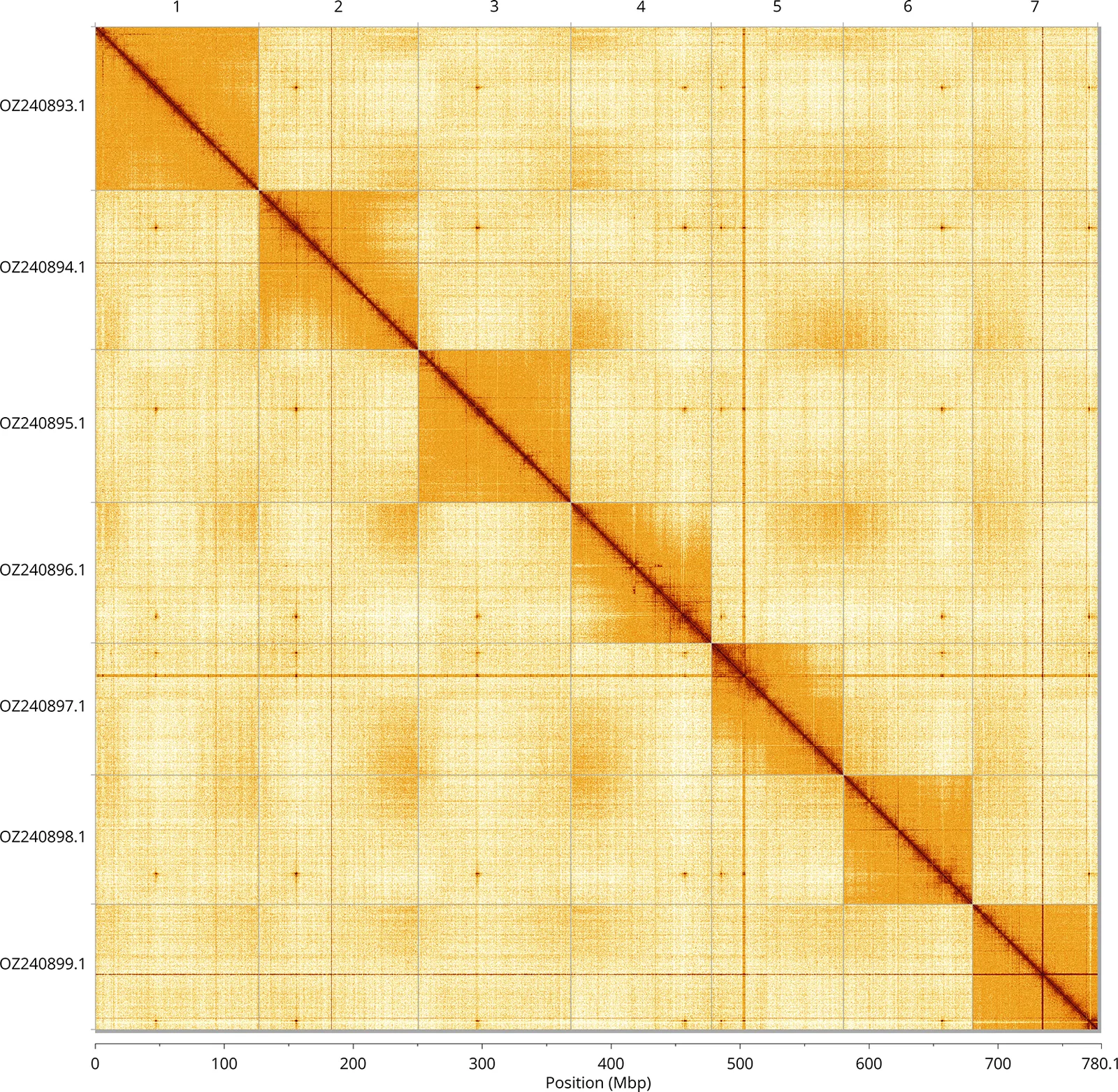
Hi-C contact map of the *L. dortmanna* genome assembly. Assembled chromosomes are shown in order of size and labelled along the axes, with a megabase scale shown below. The plot was generated using PretextSnapshot.

**Figure 4.**
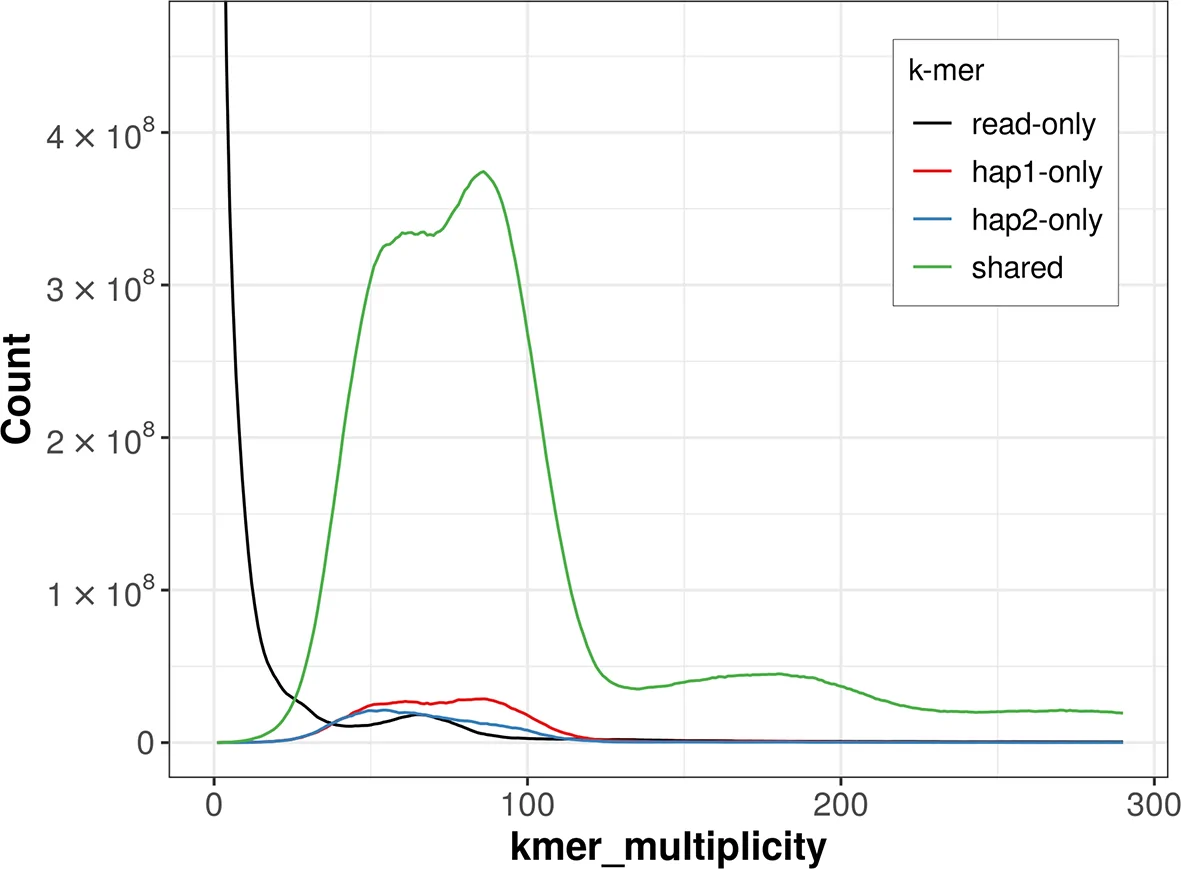
Evaluation of *k*-mer completeness using MerquryFK. This plot illustrates the recovery of *k*-mers from the original read data in the final assembly. The horizontal axis represents *k*-mer multiplicity, and the vertical axis shows the number of *k*-mers. The black curve represents *k*-mers that appear in the reads but are not assembled, while the remaining curves correspond to *k*-mers recovered in the final assembly.

**Figure 5.**
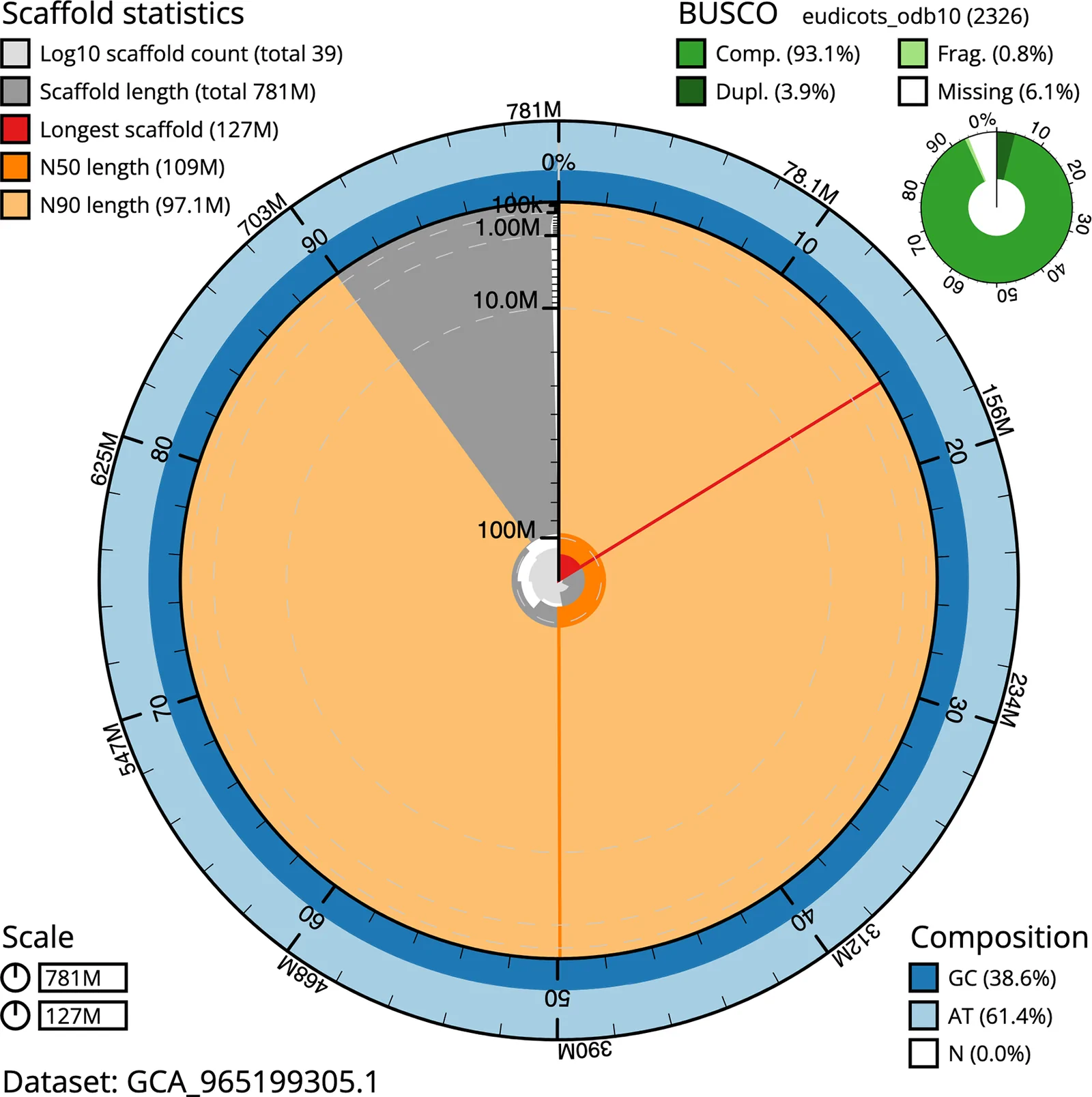
Assembly metrics for daLobDort1.hap1.1. The BlobToolKit snail plot provides an overview of assembly metrics and BUSCO gene completeness. The circumference represents the length of the whole genome sequence, and the main plot is divided into 1 000 bins around the circumference. The outermost blue tracks display the distribution of GC, AT, and N percentages across the bins. Scaffolds are arranged clockwise from longest to shortest and are depicted in dark grey. The longest scaffold is indicated by the red arc, and the deeper orange and pale orange arcs represent the N50 and N90 lengths. A light grey spiral at the centre shows the cumulative scaffold count on a logarithmic scale. A summary of complete, fragmented, duplicated, and missing BUSCO genes in the eudicots_odb10 set is presented at the top right. An interactive version of this figure can be accessed on the BlobToolKit viewer.

**Figure 6.**
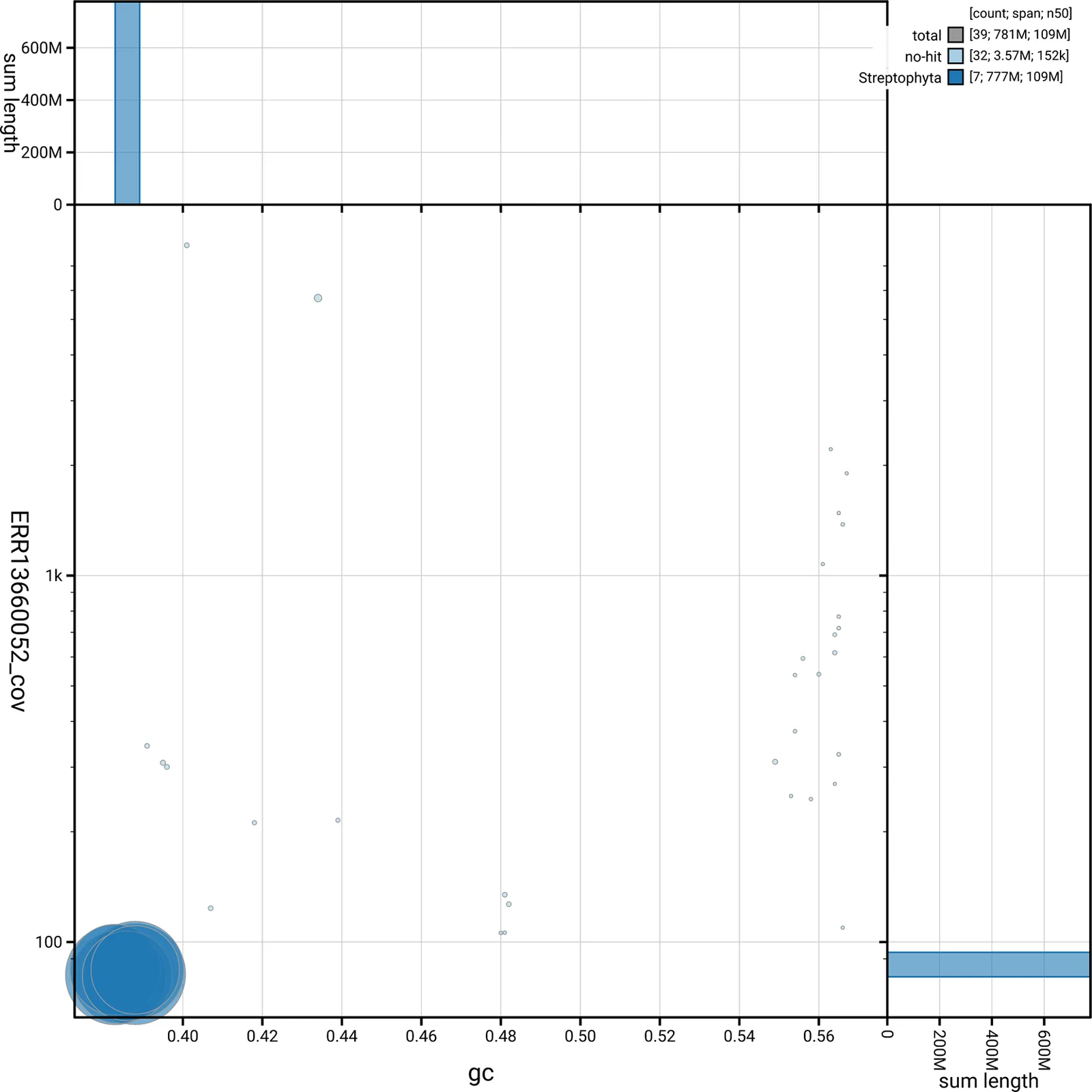
BlobToolKit blob plot for daLobDort1.hap1.1. The plot shows base coverage (vertical axis) and GC content (horizontal axis). The circles represent scaffolds, with the size proportional to scaffold length and the colour representing phylum membership. The histograms along the axes display the total length of sequences distributed across different levels of coverage and GC content. An interactive version of this figure is available on the BlobToolKit viewer.

### Assembly statistics

The genome was assembled into two haplotypes using Hi-C phasing. Haplotype 1 was curated to chromosome level, while haplotype 2 was assembled to scaffold level. The final assembly has a total length of 780.10 Mb in 37 scaffolds, with 627 gaps, and a scaffold N50 of 108.9 Mb ( Table 2).

**Table 2.** Genome assembly statistics for *L. dortmanna.*

Genome assembly	Haplotype 1	Haplotype 2
**Assembly name**	daLobDort1.hap1.1	daLobDort1.hap2.1
**Assembly accession**	GCA_965199305.1	GCA_965198955.1
**Assembly level**	chromosome	scaffold
**Span (Mb)**	780.10	756.83
**Number of chromosomes**	7	-
**Number of contigs**	664	498
**Contig N50 (Mb)**	2.05	2.48
**Number of scaffolds**	37	20
**Scaffold N50 (Mb)**	108.9	108.12
**Longest scaffold length (Mb)**	126.85	122.68
**Organelles**	Mitochondrial genome: 496.36 kb; Plastid genome: 163.59 kb	-

Most of the assembly sequence (99.63%) was assigned to 7 chromosomal-level scaffolds. These chromosome-level scaffolds, confirmed by Hi-C data, are named according to size ( Figure 3; Table 3).

**Table 3.** Chromosomal pseudomolecules in the haplotype 1 genome assembly of *L. dortmanna* daLobDort1.

INSDC accession	Molecule	Length (Mb)	GC%
OZ240893.1	1	126.85	39
OZ240894.1	2	123.50	38.50
OZ240895.1	3	118.43	39
OZ240896.1	4	108.90	38.50
OZ240897.1	5	102.26	38.50
OZ240898.1	6	100.14	38.50
OZ240899.1	7	97.12	39

The mitochondrial genome (length 496.36 kb, OZ240900.1) and plastid genome (length 163.59 kb, OZ240901.1) were also assembled. These sequences are included as contigs in the multifasta file of the genome submission and as standalone records.

### Assembly quality metrics

For haplotype 1, the estimated QV is 63.1, and for haplotype 2, 61.5. When the two haplotypes are combined, the assembly achieves an estimated QV of 62.2. The *k*-mer completeness is 91.07% for haplotype 1, 89.36% for haplotype 2, and 95.27% for the combined haplotypes ( Figure 4).

BUSCO analysis using the eudicots_odb10 reference set ( *n* = 2,326) identified 93.1% of the expected gene set (single = 89.2%, duplicated = 3.9%) for haplotype 1. The snail plot in Figure 5 summarises the scaffold length distribution and other assembly statistics for haplotype 1. The blob plot in Figure 6 shows the distribution of scaffolds by GC proportion and coverage for haplotype 1.

Table 4 lists the assembly metric benchmarks adapted from Rhie *et al.* (2021) and the Earth BioGenome Project (EBP) Report on Assembly Standards.

**Table 4.** Earth Biogenome Project summary metrics for the *L. dortmanna* assembly.

Measure	Value	Benchmark
EBP summary (haplotype 1)	6.C.Q63	6.C.Q40
Contig N50 length	2.05 Mb	≥ 1 Mb
Scaffold N50 length	108.90 Mb	= chromosome N50
Consensus quality (QV)	Haplotype 1: 63.1; haplotype 2: 61.5; combined: 62.2	≥ 40
*k*-mer completeness	Haplotype 1: 91.07%; Haplotype 2: 89.36%; combined: 95.27%	> 90%
BUSCO	Haplotype 1: C:93.1% [S:89.2%, D:3.9%], F:0.8%, M:6.1%, n:2,326; Haplotype 2: C:92.5% [S:88.5%, D:4.0%], F:0.6%, M:6.9%, n:2,326	S > 90%; D < 5%
Percentage of assembly assigned to chromosomes	99.63%	≥ 90%

*Note*: The EBP summary uses log10(Contig N50); chromosome-level (C) or log10(Scaffold N50); Q (Merqury QV). BUSCO v5.7.1 using the eudicots_odb10 lineage dataset: C = complete; S = single-copy; D = duplicated; F = fragmented; M = missing; n = orthologues.

**Table 5.** Software versions and sources used for *L. dortmanna.*

Software	Version	Source
BLAST	2.14.0	https://ftp.ncbi.nlm.nih.gov/blast/executables/blast+/
BlobToolKit	4.4.5	https://github.com/genomehubs/blobtoolkit
blobtk	0.5.1	https://github.com/genomehubs/blobtk
BUSCO	5.7.1	https://gitlab.com/ezlab/busco
bwa-mem2	2.2.1	https://github.com/bwa-mem2/bwa-mem2
DIAMOND	2.1.8	https://github.com/bbuchfink/diamond
fasta_windows	0.2.4	https://github.com/tolkit/fasta_windows
FastK	1.1	https://github.com/thegenemyers/FASTK
GenomeScope2.0	2.0.1	https://github.com/tbenavi1/genomescope2.0
Gfastats	1.3.6	https://github.com/vgl-hub/gfastats
Hifiasm	0.19.8-r603	https://github.com/chhylp123/hifiasm
HiGlass	1.13.4	https://github.com/higlass/higlass
MerquryFK	1.1.0-c1	https://github.com/thegenemyers/MERQURY.FK
Minimap2	2.24-r1122; 2.28-r1209	https://github.com/lh3/minimap2
Oatk	0.9	https://github.com/c-zhou/oatk
MultiQC	1.14; 1.17 and 1.18	https://github.com/MultiQC/MultiQC
Nextflow	23.10.0; 24.10.4	https://github.com/nextflow-io/nextflow
PretextSnapshot	0.0.6	https://github.com/sanger-tol/PretextSnapshot
PretextView	1.0.3	https://github.com/sanger-tol/PretextView
samtools	1.17; 1.18; 1.2; 1.21; 1.6	https://github.com/samtools/samtools
sanger-tol/ascc	0.1.0	https://github.com/sanger-tol/ascc
sanger-tol/blobtoolkit	v0.7.1	https://github.com/sanger-tol/blobtoolkit
sanger-tol/curationpretext	1.4.2	https://github.com/sanger-tol/curationpretext
Seqtk	1.4-r122	https://github.com/lh3/seqtk
Singularity	3.9.0	https://github.com/sylabs/singularity
TreeVal	1.1.1	https://github.com/sanger-tol/treeval
YaHS	1.2a.2	https://github.com/c-zhou/yahs

The EBP metric calculated for haplotype 1 is **6.C.Q63**, meeting the recommended 6.C.Q40 reference standard.

## Author information

• Members of the Royal Botanic Gardens Kew Genome Acquisition Lab
• Members of the Plant Genome Sizing Collective
• Members of the Darwin Tree of Life Barcoding collective
• Members of the Wellcome Sanger Institute Tree of Life Management, Samples and Laboratory team
• Members of Wellcome Sanger Institute Scientific Operations – Sequencing Operations
• Members of the Wellcome Sanger Institute Tree of Life Core Informatics team
• Members of the Tree of Life Core Informatics collective
• Members of the Darwin Tree of Life Consortium

## Wellcome Sanger Institute – Legal and Governance

The materials that have contributed to this genome note have been supplied by a Darwin Tree of Life Partner. The submission of materials by a Darwin Tree of Life Partner is subject to the **‘Darwin Tree of Life Project Sampling Code of Practice’**, which can be found in full on the Darwin Tree of Life website. By agreeing with and signing up to the Sampling Code of Practice, the Darwin Tree of Life Partner agrees they will meet the legal and ethical requirements and standards set out within this document in respect of all samples acquired for, and supplied to, the Darwin Tree of Life Project. Further, the Wellcome Sanger Institute employs a process whereby due diligence is carried out proportionate to the nature of the materials themselves, and the circumstances under which they have been/are to be collected and provided for use. The purpose of this is to address and mitigate any potential legal and/or ethical implications of receipt and use of the materials as part of the research project, and to ensure that in doing so we align with best practice wherever possible. The overarching areas of consideration are:

• Ethical review of provenance and sourcing of the material
• Legality of collection, transfer and use (national and international)

Each transfer of samples is further undertaken according to a Research Collaboration Agreement or Material Transfer Agreement entered into by the Darwin Tree of Life Partner, Genome Research Limited (operating as the Wellcome Sanger Institute), and in some circumstances, other Darwin Tree of Life collaborators.

## Data Availability

European Nucleotide Archive: Lobelia dortmanna. Accession number PRJEB79878; https://identifiers.org/ena.embl/PRJEB79878. The genome sequence is released openly for reuse. The *L. dortmanna* genome sequencing initiative is part of the Darwin Tree of Life Project ( PRJEB40665) and the Sanger Institute Tree of Life Programme ( PRJEB43745). All raw sequence data and the assembly have been deposited in INSDC databases. The genome will be annotated using available RNA-Seq data and presented through the Ensembl pipeline at the European Bioinformatics Institute. Raw data and assembly accession identifiers are reported in Tables 1 and 2. Pipelines used for genome assembly at the WSI Tree of Life are available at https://pipelines.tol.sanger.ac.uk/pipelines. Table 5 lists software versions used in this study.
