# Supplementary material for: The genome sequence of Water Lobelia, Lobelia dortmanna L., 1753 (Asterales: Campanulaceae)
Source: Wellcome Open Res. 2026 Oct 8;11:825. [Version 1] doi: 10.12688/wellcomeopenres.27537.1 (PMC13714417; doi:10.12688/wellcomeopenres.27537.1)
Supplement: Peer review history

# Reviewer report: Reviewer response for version 1

**DOI:** 10.21956/wellcomeopenres.30308.r173003

**Published:** 2026-10-27

**Authors:** Peng-Fei Ma

**Affiliations:** 1 Chinese Academy of Sciences, Kunming, China

In this study, the type species of the large genus *Lobelia* was sequenced with its genome assembled. As a perennial herbaceous aquatic plant, *Lobelia dortmanna* occurs widely in northern and western Europe and in North America. The genome assembly was resolved at the haplotype level with haplotype 1 scaffolded into seven chromosomes. Assessment indicated a high quality of the assembled genome. However, it should be noted that the final assembly size is much smaller than those estimated by K-mer analysis and C-value.

Are sufficient details of methods and materials provided to allow replication by others?

Yes

Is the rationale for creating the dataset(s) clearly described?

Yes

Are the datasets clearly presented in a useable and accessible format?

Yes

Are the protocols appropriate and is the work technically sound?

Yes

Reviewer Expertise:

Plant genomics and evolution

I confirm that I have read this submission and believe that I have an appropriate level of expertise to confirm that it is of an acceptable scientific standard.

# Reviewer report: Reviewer response for version 1

**DOI:** 10.21956/wellcomeopenres.30308.r173004

**Published:** 2026-10-14

**Authors:** Ruslan Kalendar

**Affiliations:** 1 University of Helsinki, Helsinki, Finland

The authors present a phased assembly of the diploid (2n = 14) genome of water lobelia, *Lobelia dortmanna*, the type species of *Lobelia*, generated within the Darwin Tree of Life project. The data comprise PacBio HiFi reads from a single ultra-low-input library (79.67 Gb; Revio), Hi-C (131.98 Gb), and RNA-seq. Haplotype 1 (780.10 Mb) has been curated to the chromosome level: seven pseudomolecules contain 99.63% of the sequence, with contig N50 2.05 Mb, scaffold N50 108.90 Mb, QV 63.1, and BUSCO completeness of 93.1%. Haplotype 2 (756.83 Mb) remains at the scaffold level. The mitochondrial (496.36 kb) and plastid (163.59 kb) genomes were also assembled.

 The scaffolding was excellent, and the Hi-C map was clean.

 Q1. The text provides a GenomeScope estimate of 915.17 Mb, but Figure 2 shows len = 905,817,673 bp (905.82 Mb); please reconcile. The flow-cytometry value is also internally inconsistent: 1.11 pg × 978 Mb/pg = 1,086 Mb, not 1,080.00 Mb (1,080 Mb corresponds to 1.10 pg). More importantly, haplotype 1 (780.10 Mb) is only 72% of the flow-cytometry estimate and 86% of the GenomeScope estimate, and haplotype 2 (756.83 Mb) is smaller still. The sentence “These estimates guided expectations for the assembly” does not address a shortfall of 300 Mb. With 57% repeats, collapsed satellite or rDNA arrays, and centromeric repeats are the obvious candidates. Please discuss this explicitly and, if possible, quantify the under-assembled repeat fraction (e.g., from read depth or RepeatExplorer/TAREAN on the reads).

 Q2. In Figure 2, the observed spectrum does not follow the fitted model. The model places the heterozygous and homozygous peaks at 37.5× and 75×, respectively, whereas the observed spectrum has a trough at 25×, a main peak at 50×, and a shoulder at 80×. The model clearly over-predicts at 25-45× and under-predicts at 50×. The full-model fit is not reported in the text, unlike in other notes in this series. Consequently, the heterozygosity (0.76%), repeat content (57.33%), and genome size from this fit are unreliable. A likely cause is uneven coverage from the PCR-amplified ultra-low-input library (see comment 3).

 Q3. The only HiFi library (DTOL13810556) was prepared with the ULI workflow from 20 ng of DNA (Qubit 2.38 ng/µL, 9.4 kb fragments) with whole-genome PCR amplification. The mean read length was short (79.67 Gb / 9.65 M reads = 8.3 kb). PCR amplification is known to cause GC- and sequence-dependent coverage bias and dropout, and short reads limit the resolution of long repeats. This plausibly explains several features of the assembly: the irregular k-mer spectrum, the modest contig N50 (2.05 Mb) with 627 gaps despite 74× coverage, the 6.1% missing BUSCOs, and the genome-size shortfall.

 Q4. Table 4 lists the BUSCO benchmark as “S > 90%; D < 5%,” but single-copy completeness is 89.2% for haplotype 1 and 88.5% for haplotype 2; therefore, neither assembly meets the criteria. The missing BUSCOs (6.1% and 6.9%) are relatively high for a chromosome-level plant assembly. Please state this openly and test whether the missing genes are absent from the genome or merely missing from the assembly. One option is to search for them in the RNA-seq data (or a transcriptome assembly). Another is to compare with the published *Lobelia seguinii* genome (Zhang et al., 2025): genes missing in both species would point to lineage-specific loss. The haplotype 2 BUSCO values should also be given in the text, not only in Table 4.

 Q5. Figure 3 shows sharp stripes that contact the whole genome at approximately 55 Mb on chromosome 2, 25 Mb on chromosome 5, and 57 Mb on chromosome 7. Such stripes typically mark high copy repeats (e.g., 45S rDNA/NOR loci or satellite arrays) or collapsed repeats. The blob plot (Figure 6) also shows unplaced high-coverage (200-2,000×), GC-rich (55-57%) scaffolds typical of rDNA. Please comment on these loci and, if possible, locate the 45S and 5S rDNA arrays, which would also be valuable for cytogenetic comparison within *Lobelia*. In addition, with 627 gaps, the assembly should not be described as “chromosomally complete” (Background).

 The assembly is scientifically valid, and the data are usable in their current form. The points raised in my report (Q1–Q5) are suggestions that would improve the presentation and the discussion of the assembly's limitations, but I consider them minor.

Are sufficient details of methods and materials provided to allow replication by others?

Yes

Is the rationale for creating the dataset(s) clearly described?

Yes

Are the datasets clearly presented in a useable and accessible format?

Yes

Are the protocols appropriate and is the work technically sound?

Yes

Reviewer Expertise:

plant genomics bioinformatics

I confirm that I have read this submission and believe that I have an appropriate level of expertise to confirm that it is of an acceptable scientific standard.
